# Hong Kong Chinese character psycholinguistic norms: ratings of 4376 single Chinese characters on semantic radical transparency, age-of-acquisition, familiarity, imageability, and concreteness

**DOI:** 10.3758/s13428-022-01928-y

**Published:** 2022-08-24

**Authors:** I-Fan Su, Yen Na Yum, Dustin Kai-Yan Lau

**Affiliations:** 1 Taipei, Taiwan; 2 Department of Special Education and Counselling, The Education University of Hong Kong, 10 Lo Ping Road, NT Ting Kok, Hong Kong; 3 Department of Bilingual and Chinese Studies, Hong Kong Polytechnic University, Hung Hom, Hong Kong

**Keywords:** Psycholinguistics norms, Cantonese, Chinese characters, Semantic radical transparency, Age-of-acquisition, Familiarity, Concreteness, Imageability

## Abstract

Several norms of psycholinguistic features of Chinese characters exist in Mandarin Chinese, but only a few are available in Cantonese or in the traditional script, and none includes semantic radical transparency ratings. This study presents subjective ratings of age-of-acquisition (AoA), familiarity, imageability, concreteness, and semantic radical transparency in 4376 Chinese characters. The single Chinese characters were rated individually on the five dimensions by 20 native Cantonese speakers in Hong Kong to form the Hong Kong Chinese Character Psycholinguistic Norms (HKCCPN). The split-half reliability and intra-class correlations testified to the high internal reliability of the ratings. Their convergent and discriminant patterns in relations to other psycholinguistic measures echoed previous findings reported on Chinese. There were high correlations for semantic radical transparency, imageability and concreteness, and moderate-to-high correlations for AoA and familiarity among subsets of items that had been collected in previous studies. Concurrent validity analyses showed convergence in predicting behavioral response times in various tasks (lexical decision, naming, and writing-to-dictation) when compared with other Chinese character databases. High predictive validity was shown in writing-to-dictation data from an independent sample of 20 native Cantonese speakers. Several objective psycholinguistic measures (character frequency, stroke number, number of words formed, number of homophones and number of meanings) were included in this database to facilitate its use. These new ratings extend the currently available norms in language and reading research in Cantonese Chinese for researchers, clinicians, and educators, as well as provide them with a wider choice of stimuli.

Availability of large-scaled normed datasets promotes open science and facilitates efficient scientific progress. Word databases for different language varieties have become important resources for researchers to conduct comparative studies and for clinicians to select appropriate assessment or treatment materials. Psycholinguistic word properties may be objectively calculated from surface features of a word (e.g., visual complexity) or derived from corpora (e.g., lexical frequency, phonological consistency), while others need human ratings (e.g., familiarity, imageability). As human ratings are more difficult to collect on a large scale, researchers would need to conduct study-specific ratings each time a new study was conceived, which leads to additional preparation time and sometimes duplicate efforts. To address this problem, we collected normed ratings for five psycholinguistic variables for 4376 single characters, representing almost all commonly encountered morphemes. To our knowledge, this is the first report of psycholinguistic ratings on semantic variables in single Cantonese Chinese characters with traditional script. Based on these data, we conducted analyses to fill several literature gaps about Chinese character reading.

## Characteristics of Cantonese Chinese

The majority of users of the Chinese language are located in Mainland China, Taiwan, and Hong Kong, but there are some differences in the Chinese varieties used in these three places. Orthographically, simplified script Chinese characters are used in Mainland China while traditional script characters are used in Taiwan and Hong Kong. Phonologically, Mandarin is used in Mainland China and Taiwan while Cantonese is used in Hong Kong. The current study was conducted in Hong Kong, where traditional Chinese characters and Cantonese are widely used. In the following, we briefly introduce the language and illustrate its main characteristics using examples and figures in the Hong Kong context. All phonetic transcriptions are represented in *jyutping*, a Romanization system developed by the Linguistic Society of Hong Kong.

In general, Chinese is morphosyllabic, in that each Chinese character corresponds to one syllable and one morpheme (Hoosain, 1992). For example, the character 球 corresponds to the syllable [kau4] and the meaning <ball>. The Chinese language, including Cantonese, is characterized by its opaque relations in terms of the mapping between the orthographic, phonological, and semantic systems. For phonology-to-semantics mapping, some characters correspond to multiple syllables and morphemes. The character 長, for example, refers to the syllable [coeng4] and the corresponding morpheme <long> or the syllable [zoeng2] and the corresponding morpheme <growth> depending on the word contexts in which the character is used. On the other hand, another type of characters corresponds to identical syllables but multiple morphemes, e.g., the character 足corresponds to the morpheme <foot> as in 足球 <football> and another morpheme <enough> as in 滿足 <fulfill>, while in both contexts, the character is phonologically realized as [zuk1].

The phonology-to-orthography mapping is also opaque in Chinese. There are over 5000 traditional characters used in Hong Kong, corresponding to about 1400 Cantonese syllables (Leung & Lau, 2010). That means on average, each syllable corresponds to more than three different morphemes and characters. For example, the syllable [coeng4] corresponds to both 長 <long> and 場 <field >. Being able to tell that the common syllable [coeng4] in [coeng4dou6] <length> and [zuk1kau4coeng4] <football field> corresponds to different morphemes is essential for fluent oral comprehension. Otherwise, one will be confused when trying to parse the meaning of multimorphemic words. One of the useful strategies to differentiate between homophonic heteronyms is to refer to their orthographic forms, i.e., the characters.

Each Chinese character is a compilation of strokes patterned in a rectangular construction. The number of strokes in a character varies, ranging from one to 32 in the traditional script. One major group of characters is called phonetic compounds (PCs), and they are composed of semantic radicals that give clues to meanings and phonetic radicals that give clues to phonology. For example, the PC character橡 /zoeng6/ [oak] contains the semantic radical 木 /muk6/ [wood] that gives clue to its meaning category and the phonetic radical 象 /zoeng6/ [elephant] that shares the same syllable with the character 橡. Studies have documented the significant roles of semantic and phonetic radicals in reading Chinese characters (e.g., Lee et al., 2006; Perfetti & Tan, 1998; Zhou & Marslen-Wilson, 1999; Yum & Law, 2019; Yum et al., 2014; Wang et al., 2017).

Previous works on two-character and multi-character words (Sun et al., 2018; Tsang et al., 2018; Tse et al., 2017) have highlighted the influence of single-character properties in word recognition as a whole. Properties of single Chinese characters used in different places have been reported in megastudies (e.g., mainland China: Cai et al., 2021; Liu et al., 2007; Sun et al., 2018; Tsang et al., 2018; Taiwan: Chang et al., 2016; Chang & Lee, 2020; Singapore: Sze et al., 2014). These studies usually reported behavioral performance (naming or lexical decision) predicted by various psycholinguistic properties in a large number of Chinese characters. Two previous studies investigated psycholinguistic properties of Chinese words in the Hong Kong context with Cantonese native readers: a megastudy examined lexical frequency, semantic transparency, and phonological consistency in Chinese word reading (Tse et al., 2017) and another study reported norms for affective and lexico-semantic variables (Yee, 2017). However, both studies focused on two-character words, instead of single characters. This left a research gap in psycholinguistic ratings on semantic variables in single Cantonese Chinese characters with traditional script that we sought to fill in the current study.

## Semantic radical transparency

The roles of semantic and phonetic radicals in the processing of Chinese characters have been well-documented in the literature (e.g., Chen & Weekes, 2004; Feldman & Siok, 1997; Perfetti & Tan, 1998; Taft & Zhu, 1997; Zhou & Marslen-Wilson, 1999). A few recent studies further suggested that semantic radicals may contribute more than phonetic radicals to the recognition of Chinese characters (e.g., Ho et al., 2003; Wang et al., 2017). Studies that investigated semantic radicals usually observed the significance of semantic radical transparency, which refers to the degree of meaning correspondence between the semantic radical and the whole character. For instance, 樹 <tree> is semantically transparent and 權 <power> is opaque although both characters have the semantic radical 木 <wood>. Chen and Weekes (2004) found a facilitative effect in accuracy and response time for semantically transparent characters, together with interactions with semantic radical combinability (number of characters sharing the semantic radical) and semantic radical consistency (proportion of semantically transparent characters sharing the semantic radical). The effect was only found in semantic categorization and not in lexical decision. On the other hand, using a lexical decision task associated with event-related potential measures, Wang et al. (2017) added that characters with high semantic radical transparency yielded significantly shorter response time, lower error rate, as well as smaller P200 and larger N400 in native Chinese readers.

Facilitatory effects for semantically transparent characters for behavioral response were also obtained among Chinese as foreign language learners using a paradigm requiring explicit meaning matching (Williams, 2013; Williams & Bever, 2010). Wong (2015) asked adult native Cantonese speakers from Hong Kong to perform a semantic categorization task. Results suggested that semantic radical transparency may be confounded with imageability, since its effect was no longer significant once imageability was controlled as a covariate. Critically, these reports have relied on a limited number of carefully selected characters for maximal contrasts, which precluded strong conclusions on the semantic radical transparency effect whether in simplified or traditional Chinese scripts. Few megastudies of Chinese character reading investigated features associated with the semantic radical. Reliable ratings of semantic radical transparency would facilitate further research to clarify and extend these findings.

## Concreteness and imageability

Even in the small-scale ratings used in previous research, it has been repeatedly reported that imageability and concreteness covary with semantic radical transparency (Bi et al., 2007; Wong, 2015). Imageable words are lexical items arousing a sensory experience, such as a mental picture or sound, while concrete words refer to objects, living beings, actions, and materials that can be experienced by the senses (Barca et al., 2002; Juhasz & Yap, 2013). In previous Chinese studies, both imageable and concrete characters facilitated processing (e.g., Chen & Peng, 1998; Liu et al., 2007) and are highly correlated (Liu et al., 2007; Wang et al., 2020). However, Paivio (2013) argued that the two variables are conceptually different in that imageability can only be inferred from subjective experience, and thus imageability may capture a higher degree of individual difference in word processing. Bi et al. (2007) compared characters with high and low semantic radical transparency in a meaning definition task in a patient with dementia, however, the selected transparent characters were significantly more imageable and more concrete than the opaque characters. They then analyzed the effects of these variables on the patient’s character meaning definition using logistic regression, showing that only imageability remained a significant predictor, while semantic radical transparency and concreteness did not show independent effects. In line with this finding, other studies have found that imageability predicted lexical processing better than concreteness (e.g., Marcel & Patterson, 1978; Richardson, 1975). The distinction between concreteness and imageability is a long-standing methodological issue because the conceptual difference is subtle, and researchers may use them interchangeably. As most previous ratings were collected from different raters, rating instructions may not be understood as intended. Instead, if ratings were given by the same group of raters, this may lead to better conceptual differentiation of the two properties and thus more accurate evaluation according to the instructions.

## Age-of-acquisition and subjective familiarity

A number of studies have shown that print and oral lexical frequency, age-of-acquisition (AoA), and subjective familiarity are related but distinct measures (Stadthagen-Gonzalez & Davis, 2006; Zevin & Seidenberg, 2002). AoA was shown to be a significant predictor in lexical processing across languages, independent from lexical frequency (e.g., Brysbaert & Ghyselinck, 2006; Cai et al., 2021; Chang & Lee, 2020; Juhasz, 2005; Lau et al., 2019; Yum & Law, 2019). The measurements of AoA are typically based on participants’ recall of the age at which the meaning and pronunciation of a word are acquired. Although objective AoA can also be derived from child language corpora or published school textbooks (e.g., Cai et al., 2021; Shu et al., 2003), such data are not readily available, while subjective ratings are reliable estimates of the actual age at which a word was acquired (Gilhooly & Gilhooly, 1980; Morrison et al., 1997; Xu et al., 2021). Previous studies have reported a negative correlation between AoA and imageability/concreteness (Bird et al., 2001; Kolbeneva & Alexandrov, 2016; Liu et al., 2007; Stadthagen-Gonzalez & Davis, 2006), while familiarity is positively correlated with imageability/concreteness (Liu et al., 2007; Stadthagen-Gonzalez & Davis, 2006; Yee, 2017).

AoA and familiarity are both common variables in psycholinguistic and memory research, however, as mentioned, large-scale ratings of these variables in Hong Kong readers are not available. In some studies, these potential confounding variables are left unmatched. Alternatively, some studies involving participants in Hong Kong selected stimuli based on measures derived from samples in mainland China or Taiwan. While this choice can be understood on practical ground, it may not be appropriate to assume that properties of Chinese characters from places with spoken Mandarin are equivalent or transferrable to Cantonese Chinese. Divergent lexical uses occur naturally in different geographic regions. For example, Cantonese words have a higher tendency to be single character (鼻 <nose>), compared to the Mandarin counterparts (鼻子 <nose>). Thus, the number of words formed by single Cantonese and Mandarin characters may differ. Similarly in the phonological domain, the number of homophones in Cantonese and Mandarin are different. Educational practices also differentiate Chinese reading in Hong Kong from that in other places. Specifically, Hong Kong reading pedagogy typically relies on a “look-and-say” method without a systematic phonetic code, such as *pinyin* in mainland China or *zhuyin fuhao* in Taiwan. Furthermore, the traditional script mainly used in Hong Kong and Taiwan differed from the simplified script used in mainland China and Singapore in several ways – simplified characters may have simplified radical forms (e.g., 語 → 语), replacement of radicals with existing radicals (e.g., 聽 → 听), or characters merging with an existing character (e.g., 遊 and 游 → 游) (see Lam, 2003, for review of the simplification scheme). These differences necessitate new ratings that are appropriate for local use in Hong Kong.

## The current study

In this study, we collected ratings of the five aforementioned lexico-semantic measures from skilled Hong Kong Chinese readers to form the Hong Kong Chinese Character Psycholinguistic Norms (HKCCPN). The current study applied a within-rater approach by recruiting reliable participants who provided ratings for all variables over multiple days (about 15 h in total) in a controlled laboratory environment. Most large-scale rating studies relied on data collection from many participants, but since human ratings are subjective in nature, the data are vulnerable to variance among raters. Data collected using a within-rater approach have the advantage of capturing within-participant variance and have better interpretability due to smaller baseline differences. Keuleers et al. (2010) took the within-rater approach and reported minimal practice effects in lexical decision performance to over 14,000 Dutch words and non-words from the same participants. Thus, we did not expect that the multiple day procedure would significantly affect the reliability of the ratings.

Analyses were done to describe the distribution of the collected ratings, establish their reliability, and explore relationships between different psycholinguistic variables. We expected to replicate some previous findings, including (1) strong relationship between ratings of familiarity, AoA, and character frequency (e.g., Bird et al., 2001; Cai, et al., 2021; Kolbeneva & Alexandrov, 2016; Lau et al., 2019; Liu et al., 2007; Stadthagen-Gonzalez & Davis, 2006; Xu et al., 2021; Yum & Law, 2019; Zevin & Seidenberg, 2002), (2) strong relationship between concreteness and imageability (e.g., Liu et al., 2007; Wang et al., 2020), (3) moderate but significant relationship between semantic radical transparency and imageability/concreteness (Bi et al., 2007; Wong, 2015), and (4) negative relationship between AoA and imageability/concreteness (Bird et al., 2001; Kolbeneva & Alexandrov, 2016; Liu et al., 2007; Stadthagen-Gonzalez & Davis, 2006). We also predicted discriminant validity between the current ratings and two semantic variables (number of words formed and number of meanings), an orthographic variable (number of stroke), and a phonological variable (number of homophones). Concurrent validity of the HKCCPN ratings were shown by comparing our dataset with other publicly available datasets of single Chinese characters, while predictive validity was established by using the norms to predict writing-to-dictation performance in an independent sample of native Cantonese speakers.

## Method

### Participants

A total of 20 undergraduate students (gender-balanced; mean age = 20.2 years, S.D. = 1.6, range = 18–24 years) were recruited for the rating tasks. A separate group of 20 undergraduate students (mean age = 20.5 years, S.D. = 1.5, range = 19–24 years) were recruited for the writing-to-dictation task. All participants were native Cantonese speakers who used Cantonese as their dominant language for daily communication^1^ and had received mainstream education in Hong Kong since first-level of kindergarten. All of them attained level 4 or above in the composite Chinese grade in the Hong Kong Diploma of Secondary Education Examination. This public examination is taken for university entrance in Hong Kong, using standards-referenced grading with annual calibration exercises with 1 being the lowest level and 5 the highest, and level 3 is typically required for admissions for undergraduate studies. All participants reported normal or corrected-to-normal vision and no history of cognitive or learning disabilities. They also reported no formal psychology or linguistics training, and they were paid for their participation in the study.

### Stimuli

A total of 4376 traditional Chinese characters, consisting of 3327 PCs and 1049 non-phonetic compounds (nonPCs), were included in the rating experiment. Characters were categorized into PCs or nonPCs according to the Shuowen Jiezi Zhu (Xu, 1963) dictionary, which reported the origins of Chinese characters. Character frequency, the per million count of appearances of the character, and number of homophones, the number of different characters sharing the same syllable, were calculated from the Hong Kong Corpus of Chinese Newspapers (Leung & Lau, 2010). The corpus was formed from 123,677 news articles published by the eight most popular Chinese newspaper publishers in Hong Kong and contained approximately 7.6 million characters. Number of words formed is defined as the number of different multi-character words containing the character, independent of the character position in the multi-character word (Liu et al., 2007; Tsang et al. 2018). This variable and the number of meanings associated with each character (Liu et al., 2007; Tsang et al., 2018) were based on the Chinese Character Database: With Word-Formations Phonologically Disambiguated According to The Cantonese Dialect (Kwan et al., 2012).

In the writing-to-dictation task, a subset of 3051 Chinese PCs were selected from the 3126 PCs with semantic radical transparency ratings. The unselected PCs were infrequent characters mainly used in names of people (e.g., 堃, 晞, 錚). Although it was possible to use people’s names as the word contexts to elicit responses from our participants, the names would likely vary in familiarity to participants, therefore, we excluded these characters in the writing-to-dictation task.

### Procedure

#### Rating tasks

In the current study, each participant was instructed to give the ratings of *imageability*, *AoA*, *concreteness*, and *familiarity* of all 4376 target characters as well as the ratings of *semantic radical transparency* of 3126 PCs^2^, in five rating tasks. All participants followed the same rating task order of *imageability → AoA → concreteness → familiarity → semantic radical transparency.* The order was designed to separate the three ratings that were based on semantic characteristics (i.e., imageability, concreteness and semantic radical transparency) by using the two ratings that were based on lexical exposure (i.e., AoA and familiarity). This arrangement avoided the consecutive order of tasks with similar rating basis to minimize the potential carryover effect between tasks.

Rating data were collected using SurveyMonkey. The target characters were first randomly divided into 22 groups, each containing 198 or 199 targets (for semantic radical transparency, characters were divided into 16 groups, each containing 195 to 196 targets). For each task, the corresponding 22 groups of targets were then uploaded to SurveyMonkey to construct 22 individual surveys, in which one question item was created for each target character. For each constructed survey, the “one question at a time” and “question randomization” options were used to ensure the random order of presentation of each target during the task. For each participant, a random survey order was generated for each rating task and a research assistant was responsible for ensuring each participant followed the corresponding generated survey order. Each participant was tested individually in a quiet room using a desktop computer. The instructions of each rating task were adapted from those used by Barca et al. (2002) (see the Appendix). For each rating item, a seven-point scale was used. Each participant attended ten sessions to complete all the tasks. Short breaks were given upon the completion of each survey during the sessions. The average duration of each session was 1.5 h.

#### Writing-to-dictation task

A writing-to-dictation task was conducted where the participants were instructed to write their response on a Wacom Intuos Pro Large digitizer after hearing an auditory presentation of the target Chinese character. Each participant was assessed individually in a quiet room. The Ductus software (Guinet & Kandel, 2010) was used to control the display of auditory stimulus and collect handwriting output of each trial. Prior to the task, two practice trials using very high-frequency characters were given to ensure that the participants understood the instructions. In each trial, a disyllabic word context of the target character was given to avoid confusion (e.g., “「背包」嘅「背」字” [the ‘back’ in ‘backpack’]). No feedback on accuracy was given. The 3051 characters were pre-randomized and divided into ten blocks, each containing 294-310 trials, for each participant. The participants completed each block on separate days. Within each block, three short breaks were given. The total time required was about 12–15 h. Accuracy of each trial was scored offline by two research assistants.

## Results and discussion

### Database & descriptive statistics

The database was developed with raw values of 4376 characters, each rated by 20 participants on five variables. Individual outlying trials with ratings 2.5 SDs below or above the mean rated by all participants were excluded. The number of outliers comprised 1.33% of the data for AoA, 2.48% for familiarity, 0.88% for imageability, 1.09% for concreteness, and 1.08% for semantic radical transparency. After eliminating the outliers, each character retained at least 18 ratings in each dimension. The database includes the full list of all 4376 characters and their corresponding means and *SD*s of AoA, familiarity, imageability, concreteness, and semantic radical transparency ratings. Some additional variables were included for convenience of use: character frequency, number of strokes, number of homophones, number of words formed, and number of meanings. Table 1 shows the descriptive statistics for all the variables included in the database.

**Table 1:** Descriptive statistics of the measures included in the Hong Kong Chinese Character Psycholinguistic Norms

Measures	Mean	SD	Median	Min	Max
AoA	4.46	1.14	4.45	1.00	7.00
FAM	5.93	0.98	6.25	1.11	7.00
IMG	3.68	1.29	3.68	1.05	6.95
CONC	3.54	1.33	3.50	1.00	6.95
SemTran	4.78	1.27	5.00	1.50	7.00
CF	220	650	29.80	0.03	19447.00
#Strokes	12.10	4.46	12	1	32
#Words formed	5.74	5.01	4	0	62
#Homophones	4.86	4.47	4	0	28
#Meanings	3.04	2.28	3	0	19

AoA, age-of-acquisition; FAM, Familiarity; IMG, imageability; CONC, concreteness; SemTran, semantic radical transparency; CF, character frequency measured as occurrences per million; #, number of.

**Fig. 1:**
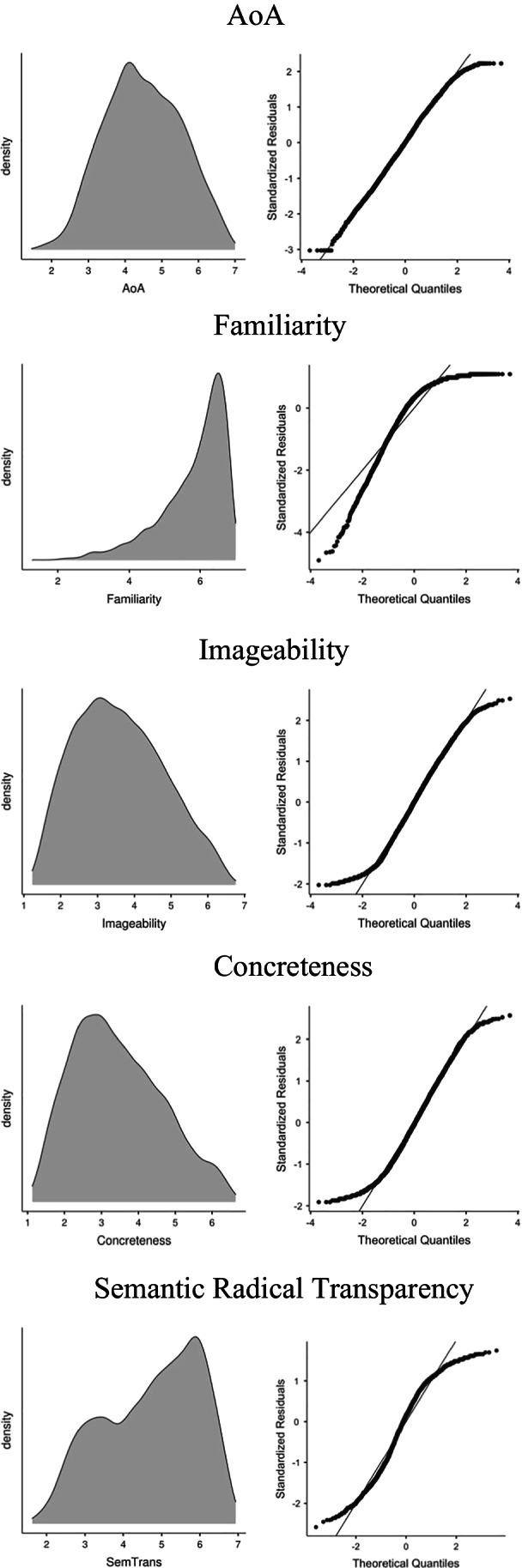
Distribution (*left*) and Q-Q plots (*right*) of the Hong Kong Chinese Character Psycholinguistic Norms measures

Table 2 and Fig. 1 show the distribution of the mean ratings of AoA, familiarity, imageability, concreteness, and semantic radical transparency. The distribution deviated significantly from a normal distribution for all measures based on the Shapiro–Wilk test: all *W* > 0.82, *p* < .001. However, as the Shapiro–Wilk test is sample-size dependent and less sensitive when applied to large datasets, skewness and kurtosis distributions and Q-Q plots were further examined. All measures showed skewness and kurtosis within the accepted range (i.e., values greater than –1 and less than 1), except for familiarity. Kurtosis was the highest for familiarity at 1.82, indicating a relatively peaked distribution compared with the normal model. Familiarity was also left skewed with many words appearing highly familiar to the raters. Most characters may appear familiar to participants because of three reasons. First, all participants were receiving tertiary education with good Chinese language ability as indicated by language examination scores, second, the items were selected from newspapers, which are unlikely to use obscure characters, and third, familiarity was rated after seeing these items for three times in other rating tasks.

**Table 2:** Skewness and kurtosis of the Hong Kong Chinese Character Psycholinguistic Norms ratings

Measures	Skewness	SE Skewness	Kurtosis	SE Kurtosis	Shapiro–Wilk *W*	Shapiro–Wilk *p*
AoA	–0.14	0.04	–0.395	0.07	0.995	< .001
FAM	–1.39	0.04	1.820	0.07	0.862	< .001
IMG	0.08	0.04	–0.713	0.07	0.986	< .001
CONC	0.24	0.04	–0.695	0.07	0.981	< .001
SemTran	–0.37	0.04	–0.944	0.09	0.956	< .001

AoA, age-of-acquisition; FAM, Familiarity; IMG, imageability; CONC, concreteness; SemTran, semantic radical transparency.

### Reliability

To evaluate the collected ratings’ internal reliability, split-half correlations were calculated for the five rated dimensions (i.e., AoA, familiarity, imageability, concreteness, and semantic radical transparency). Participants were divided into two groups of even- and odd-numbered participants, and the averaged estimates were calculated for each group for all characters. All ratings from the odd and even groups were found to correlate very highly for all dimensions (all *r*^*2*^s > .86, *p’*s < .001), and gave very high split-half reliability estimates (Kuperman et al., 2012) between .92 and .97 as shown in Table 3.

**Table 3:** Split-half correlation (Pearson’s *r*) and reliability between odd and even raters

Measures	*r*^*2*^	95% CI			*p*	*Reliability*
AoA	.94	.936	–	.943	< .001	.97
FAM	.91	.906	–	.916	< .001	.95
IMG	.86	.855	–	.870	< .001	.92
CONC	.92	.915	–	.924	< .001	.96
SemTran	.90	.887	–	.901	< .001	.95

*Note*. AoA, age-of-acquisition; FAM, Familiarity; IMG, imageability, CONC, concreteness; SemTran, semantic radical transparency

Intra-class correlation coefficients (ICCs; Bartko, 1966; Shrout & Fleiss, 1979) were also calculated for the five dimensions separately using average measures, two-way random effects model, and absolute agreement definition. Adopting the same approach as Guasch et al. (2016), an ICC was obtained for each item (i.e., character) rated by the participants. Subsequently, the mean ICC was calculated by averaging the ICCs of all the items in the dimension of interest. The mean and standard deviation of the ICCs for each dimension are shown in Table 4. Overall, very high to excellent ICCs were obtained for all five dimensions (all ICCs > .85), indicating high inter-rater reliability and consistency in our samples. As the ICC calculation would exclude items with missing data (i.e., individual outlier ratings excluded from the data trimming procedure), ICCs on the raw data were also calculated. The ICCs were higher when items with outlier ratings were included (all ICCs > .90), suggesting that the items that were not rated by all participants did not adversely affect the overall reliability.

**Table 4:** Intra-class correlation coefficients (ICCs) of rated dimensions

Measures	Mean ICC	95% CI	*F* test with true value 0			
			Value	*df*1	*df*2	*p*
AoA	.943	.932–.952	23.5	285	3218	< .001
FAM	.876	.846–.901	12.2	150	2420	< .001
IMG	.847	835–859	7.2	1757	3592	< .001
CONC	.871	843–892	10.7	214	3458	< .001
SemTran	.887	863–906	12.0	220	2501	< .001

AoA, age-of-acquisition; FAM, Familiarity; IMG, imageability, CONC, concreteness; SemTran, semantic radical transparency.

Overall, the findings from the split-half reliability and ICC analyses indicated that ratings for AoA, familiarity, imageability, concreteness, and semantic radical transparency were highly reliable and rated similarly across different participants. In both analyses, AoA showed the highest internal reliability and imageability the lowest (although still very high). The imageability results echoed previous observations that as imageability was inferred from arousing a subjective sensory experience, it captures a greater degree of individual differences as compared to other measures such as concreteness and AoA (Juhasz & Yap, 2013; Paivio, 2013).

There were two potential sources of practice or order effects in the rating tasks – one due to the fixed order of the five variables and the other due to the length of the tasks resulting in a maximum of 22 sessions for each variable. To explore whether the orders systematically affected the ratings’ reliability, we conducted a linear mixed-effects regression (LMER) model examining both potential order effects together using the *lme4* package (Bates et al., 2015) in R Version 3.5.3 (R development core team, 2019). First, we calculated a difference score between each individual trial’s rating and the trimmed average of the item, and the absolute value of this difference score was used as the dependent variable. As the distribution of the difference score was positively skewed, a square root transformation was applied to obtain a normal distribution. The fixed effects of the model were the rating type, the session order, and their interaction. For rating type, we used a categorical variable with five levels rather than a continuous variable representing the order of the rating tasks. This was because we alternated the order based on the conceptual differences in the underlying constructs of the rating types (AoA and familiarity are based on exposure, while concreteness, imageability, and semantic radical transparency are based on character meaning), so we expected that the order effect would not be linear. The continuous variable of session order was centered and z-transformed to reduce collinearity and facilitate the comparison of effect sizes, respectively. The random effects included the random intercept by participants and the random slope of variables by participants. The model was estimated using the restricted maximum likelihood method in the *lmerTest* package (Kuznetsova et al., 2017), and *p* values were calculated with the Satterthwaite approximation. Post hoc pairwise comparisons with *p* value adjustments using the Tukey method were implemented using the *emmeans* package (Lenth, 2021).

Descriptive statistics of the difference scores are shown in Table 5. The following model results were summarized by Type III ANOVA. A significant main effect of rating type was found, *F*(4,19) = 17.5, *p* < .001. Pairwise comparisons indicated that AoA had lower difference scores than concreteness, imageability, and semantic radical transparency (*p*s < .001). Familiarity showed a similar pattern with lower difference scores than concreteness (*p* = .001), imageability (*p* < .001), and semantic radical transparency (*p* = .009). AoA did not differ from familiarity (*p* = .997), and the other three variables did not differ among themselves (*p*s > .394). The main effect of session order was significantly negative, *F*(1, 409374) = 15.7, *p* < .001, indicating that raters tended to converge to more consistent ratings with increased practice. There was a significant interaction between rating type and session order, *F*(4, 409374) = 8.96, *p* < .001. In the estimated marginal means shown in Table 5, the 95% CIs of AoA and concreteness did not include the value 0, while those of the other variable types did, indicating that the negative session order main effect was driven by AoA and concreteness.

**Table 5:** Descriptive statistics of the absolute differences scores (square root transformed)

Measures	Absolute difference	SE	95% CI		Session order	SE	95% CI	
AoA	0.864	0.029	[0.81	0.92]	–0.009	0.001	[–0.011	–0.006]
FAM	0.880	0.029	[0.82	0.94]	0.000	0.001	[–0.002	0.003]
IMG	1.108	0.024	[1.06	1.16]	0.001	0.001	[–0.001	0.004]
CON	1.063	0.030	[1.01	1.12]	–0.005	0.001	[–0.008	–0.003]
SemTran	1.042	0.036	[0.97	1.11]	–0.002	0.002	[–0.006	0.003]

AoA, age-of-acquisition; FAM, Familiarity; IMG, imageability, CONC, concreteness; SemTran, semantic radical transparency.

Model results suggested that the ratings based on exposure (i.e., AoA and familiarity) had higher agreements among raters than ratings that were based on semantic characteristics (i.e., concreteness, imageability, and semantic radical transparency). This was not surprising since raters could assess AoA and familiarity based on the whole character, but may assess different semantic features when considering concreteness, imageability, or semantic radical transparency as single Chinese characters are polysemous. This pattern justified our decision to alternate the rating variables to separate the exposure-based and semantic-based variables to reduce potential carryover effects. Overall, session order either had negligible influence or that raters became more reliable and produced ratings that had higher agreements in later sessions, supporting the use of a within-rater approach to obtain more reliable ratings.

### Convergent and discriminant validity

Table 6 shows the correlations between AoA, familiarity, imageability, concreteness and semantic radical transparency and a selection of lexical variables: number of strokes, character frequency, number of homophones, number of words formed, and number of character meanings. All the subjective ratings correlated significantly with each variable, except character imageability with number of strokes. However, with large N size datasets, very small correlation coefficients can be statistically significant, and therefore the derived *p* values do not provide relevant information on how strongly the measures are correlated. Instead, we described the magnitude of the correlations by adopting suggested interpretations of the correlation coefficients by Schober et al. (2018) – negligible (.00 – .09), weak (.10 – .39), moderate (.40 – .69), strong (.70 – .89) and very strong (.90 – 1.00) correlations.

**Table 6:** Pearson’s *r* correlations and [95% CI] between subjective ratings from the Hong Kong Chinese Character Psycholinguistic Norms and selected lexical variables

Variable	AoA	Fam	IMG	CONC	SemTran*
AoA	+1.00	–	–	–	–
FAM	**–.84** [–.85, – .83]	+1.00	–	–	–
IMG	**–.49** [–.51, –.46]	**+.48** [+.46, +.50]	+1.00	–	–
CONC	**–.49** [–.50, –.46]	**+.46** [+.43, +.49]	**+.92** [+.92, +.93]	+1.00	–
SemTran*	–.10 [–.14, –.07]	+.10 [+.07, +.14]	**+.48** [+.45, +.51]	**+.48** [+.45, +.50]	+1.00
CF	**–.75** [–.76, –.74]	**+.73** [+.72, +.75]	+.18 [+.16, +.21]	+.18 [+.15, +.21]	–.15 [–.18, –.12]
#Strokes	+.32 [+.29, +.34]	–.20 [–.23, –.17]	+.02^n.s.^ [–.01, .05]	+.05 [+.02, +.08]	+.07 [+.04, +.11]
#Words Formed	**–.42** [–.45, –.40]	**+.42** [+.39, +.44]	+.18 [+.15, +.20]	+.17 [+.14, +.20]	–.07 [–.11, –.05]
#Homophones	+.13 [+.10, +.16]	–.13 [–.16, –.10]	–.13 [–.16, –.10]	–.13 [–.16, –.10]	–.04 [–.07, –.01]
#Meanings	–.28 [–.31, –.25]	+.32 [+.29, +.35]	+.05 [+.02, +.08]	+.04 [+.01, +.07]	–.12 [–.16, –.08]

AoA, age-of-acquisition; FAM, Familiarity; IMG, imageability; CONC, concreteness; SemTran, semantic radical transparency; CF, log-transformed character frequency; #, number of; Correlations of moderate strength or above (>.40) are in bold.

* only 3126 PC are involved for correlations with semantic radical transparency

n.s. not significant at the *p* > .05 significance level; all other correlations are significant at the *p* < .05 level

As expected, AoA showed a strong negative correlation with familiarity (*r* = –.84) and character frequency (*r* = –.75), and moderate negative correlation with imageability (*r* = –.49), concreteness (*r* = –.49), and number of words formed (*r* = –.42). These findings agree with conclusions drawn from previous studies of AoA in Mandarin and other languages where early acquired words are highly familiar (Bird et al., 2001; Brown & Watson, 1987; Gilhooly & Gilhooly, 1980; Liu et al., 2007; Stadthagen-Gonzalez & Davis, 2006; Zevin & Seidenberg, 2002), occur more frequently (Bird et al., 2001; Brown & Watson, 1987; Cai, et al., 2021; Gilhooly & Gilhooly, 1980; Kolbeneva & Alexandrov, 2016; Liu et al., 2007; Morrison et al., 1997; Stadthagen-Gonzalez & Davis, 2006; Xu et al., 2021; Yum & Law, 2019; Zevin & Seidenberg, 2002), can form more words (Liu et al., 2007), and tend to be more concrete and easier to imagine (Bird et al., 2001; Kolbeneva & Alexandrov, 2016; Liu et al., 2007; Stadthagen-Gonzalez & Davis, 2006). Similarly, strong correlations were also observed for familiarity and frequency (*r* = .73), and moderate correlations with imageability(*r* = .48), concreteness (*r* = .46), and number of words formed (*r* = .42). Characters are perceived to be more familiar when they can form multiple words (Liu et al., 2007), are of high word frequency (Bird et al., 2001; Brown & Watson, 1987; Gilhooly & Gilhooly, 1980; Liu et al., 2007; Yee, 2017; Zevin & Seidenberg, 2002), or are concrete or imageable (Liu et al., 2007; Stadthagen-Gonzalez & Davis, 2006; Yee, 2017).

Imageability and concreteness showed very strong correlation (*r* = .92), indicating that more imageable words tend to be more concrete, while less imageable ones are less concrete. This was consistent with previous reports of imageability and concreteness in single Chinese characters in simplified script, which showed *r* values of .796 (Liu et al., 2007) and .804 (Wang, et al., 2020). Semantic radical transparency exhibited moderate correlations with imageability (*r* = .48) and concreteness (*r* = .48), suggesting that characters that were more highly related in meaning with their sublexical semantic radical were also more concrete and imageable (Han et al., 2007; Wong, 2015). However, it showed negligible correlations with the other less meaning-based measures of AoA (*r* = –.10) and familiarity (*r* = .10).

With regards to the additional lexical variables, all the five rated dimensions showed expectantly negligible to weak correlations (*r*s = .02–.32) with the character’s visual complexity indexed by the number of strokes. The strongest correlation was with AoA (*r* = .32) where words learned early tend to contain fewer strokes, as also noted by Liu et al. (2007). The number of meanings associated with a character showed negligible to weak correlations (*r*s = –.28 – +.32) with all the ratings with familiarity being the strongest (*r* = .32). The positive correlation suggests that characters with more meanings tend to be perceived as being more familiar than ones with one distinct meaning. Number of words formed showed little associations with the rated imageability (*r* = .18), concreteness (*r* = .17), and semantic radical transparency (*r* = –.07), given that these semantic measures relate to meanings of the individual characters rather than their combination with other characters to form words. Lastly, the number of homophones of a character did not correlate strongly with any of the obtained subjective ratings (*r*s = –.13 – +.13), indicating that AoA, familiarity, semantic radical transparency, imageability, and concreteness are distinct from this phonological measure.

### Concurrent validity

Table 7 summarized the correlations (Pearson’s *r*) between the ratings of the current study and ratings reported in other published Chinese datasets. These external datasets were selected because they contain the largest numbers of stimuli for these ratings and are representative of how single characters are processed in their respective regions. In general, all comparisons showed moderate to strong pairwise correlations. The ratings obtained from the current study significantly correlate with those of external datasets despite the procedural differences in data collection. Hence, the concurrent validity of the data reported in the current study was supported.

**Table 7:** Pearson’s *r* correlations between the Hong Kong Chinese Character Psycholinguistic Norms and previously published subjective ratings

Study	Variables	*N*	*r*	Language medium of the experiments
Wong (2015)	Semantic radical transparency	270	.918^**^	Cantonese – traditional characters
	Imageability	302	.908^**^	
Chang et al. (2016)	Familiarity	2475	.805^**^	Mandarin – traditional characters
Chang & Lee (2020)	AoA	1272	.636^**^	Mandarin – traditional characters
	Imageability	1272	.749^**^	
Liu et al. (2007)	AoA	2169	.856^**^	Mandarin – simplified characters
	Imageability	2169	.757^**^	
	Familiarity	2169	.534^**^	
	Concreteness	2169	.819^**^	
Wang et al. (2020)	Imageability	1533	.805^**^	Mandarin – simplified characters
	Concreteness	1533	.897^**^	
	Familiarity	1533	.616^**^	

*N*, Number of overlapping stimuli between the published study and the current study

^**^ Correlation is significant at the *p* < .01 level

We observed no specific trend that the degrees of correlation, with Pearson’s *r* ranging from .53 to .86, may be related to similarities between language medium or script when the current study was compared with studies conducted using Mandarin-traditional characters and Mandarin-simplified characters. However, the highest correlations were observed between the current study’s imageability and semantic radical transparency ratings and those in the Wong (2015), which, like the current study, was also conducted in native Cantonese speakers using traditional characters. Although Wong’s dataset contained relatively few ratings (270 single Chinese characters containing only clearly meaningful semantic radicals), the high correspondence supported that a unique set of ratings is needed for Cantonese-traditional characters to serve as reference for future studies conducted in this language medium.

A separate set of correlations (Pearson’s *r*) examined the associations between the ratings of the current study and behavioral performance (naming, lexical decision, dictation) reported in three recently published Chinese datasets (Chang et al., 2016; Chang & Lee, 2020; Wang et al., 2020). These were calculated with the aim of examining whether the patterns of significant associations correspond to external datasets. These external datasets were selected based on their numbers of stimuli (*N* > 1000) with behavioral data in single character processing tasks. However, note that there was no published Cantonese dataset to our knowledge that fulfilled this criterion to be included in the analysis. Table 8 summarizes the comparisons of the reported *r* values and those obtained in the current study. Consistent with the findings in Chang et al. (2016) and Chang and Lee (2020), shorter RT and higher accuracies in naming and lexical decision tasks were associated with higher imageability, higher concreteness, higher familiarity, and lower AoA ratings of our dataset. Meanwhile, higher writing-to-dictation accuracy and shorter RT were associated with higher imageability, higher concreteness and higher familiarity ratings in both Wang et al. (2020)’s and our datasets. When the *r* values were categorized by descriptors suggested by Schober et al. (2018), our familiarity ratings correlate moderately with naming RT and weakly with naming accuracy. AoA correlates moderately with lexical decision RT and naming RT, and imageability correlates weakly with lexical decision RT and naming RT. Writing-to-dictation accuracy and latency correlates moderately with familiarity but weakly with imageability and familiarity. The degrees of associations were qualitatively identical between the HKCCPN and those of the three external studies. Associations of our ratings and previous ratings on behavioral measures were remarkably comparable, supporting the concurrent validity of the current ratings across tasks.

**Table 8:** Associations of behavioral measures with psycholinguistic variables in previous studies (r1) and the Hong Kong Chinese Character Psycholinguistic Norms (r2)

Study	Behavioral measures	Variables	*N*	r1	r2
Chang et al. (2016)	Naming RT	Familiarity	2477	–.669**	–.546**
	Naming Acc	Familiarity	2477	.393**	.365**
Chang & Lee (2020)	LDT RT	AoA	1274	.454**	.596**
		Imageability	1274	–.140**	–.142**
	Naming RT	AoA	1274	.440**	.513**
		Imageability	1274	–.208**	–.167**
Wang et al. (2020)	Dictation Acc	Imageability	1533	.102**	.092**
		Familiarity	1533	.560**	.627**
		Concreteness	1533	.011	.087**
	Dictation RT	Imageability	1533	–.137**	–.133**
		Familiarity	1533	–.606**	–.657**
		Concreteness	1533	–.077**	–.134**

N, Number of stimuli overlapped between the published and the current experiment; Naming RT, response time in character naming task; Naming Acc, accuracy in character naming task; LDT RT, response time in lexical decision task; Dictation Acc, accuracy in writing-to-dictation task; Dictation RT, latency in writing-to-dictation task; r1, Pearson’s *r* correlation reported in the external study; r2, Pearson’s *r* correlation obtained in the current study

** Correlation is significant at the *p* < .01 level

### Predictive validity

To examine the predictive validity of the present ratings, we obtained and analyzed the relationship between our ratings and writing performance of 20 independent native Cantonese readers for 3051 PC characters. First, the Pearson’s *r* correlations of the different psycholinguistic variables and the writing-to-dictation accuracy were computed. Results in Table 9 indicated that the accuracy of writing-to-dictation negatively correlated with AoA, and positively correlated with imageability, concreteness, familiarity, and semantic radical transparency (all *ps* < .01). The pattern of the results echoed previous findings regarding writing-to-dictation accuracy (e.g., Lau, 2021; Wang et al., 2020) and follow the predictions of the dual route account of writing-to-dictation of Chinese (Lau, 2021; Weekes et al., 2006), which suggests that writing-to-dictation of Chinese is governed by the direct lexical and the lexical-semantic pathways.

**Table 9:** Pearson’s *r* correlations between the psycholinguistic variables and average writing-to-dictation accuracy of the selected phonetic compound characters

	Acc	AoA	FAM	IMG	CON
AoA	–.755^**^				
FAM	.764^**^	–.849^**^			
IMG	.357^**^	–.505^**^	.493^**^		
CON	.336^**^	–.489^**^	.461^**^	.916^**^	
SemTran	.062^**^	–.083^**^	.069^**^	.476^**^	.473^**^

Acc, average accuracy of writing-to-dictation; AoA, age-of-acquisition; FAM, Familiarity; IMG, imageability; CONC, concreteness; SemTran, semantic radical transparency

^**^ *p* < .01

To examine the simultaneous effects of different psycholinguistic variables in predicting writing-to-dictation accuracy, a generalized linear mixed model fit by maximum likelihood with Adaptive Gauss-Hermite Quadrature of 0 was used. The dependent variable was writing accuracy, which was binomial with correct or incorrect responses. The fixed factors included the five ratings, character frequency, number of strokes, and session order. All continuous variables were z-transformed. Random intercepts of participant and item and random slopes by participant for AoA, familiarity, semantic radical transparency, character frequency, and session order were included. The kappa value for the model was 5.45, while all variance inflation factors were between 1 and 5, indicating moderate levels of multicollinearity that did not warrant corrections. Table 10 presents the parameters of the fixed effects.

**Table 10:** Parameter estimates of the generalized linear mixed model of writing-to-dictation accuracy

Model fit: AIC = 52654; log-likelihood = –26296					
Fixed factor	β	*SE*	*Z*	*p*	
Character frequency	0.632	0.048	13.2	<.001	***
Number of strokes	–0.103	0.027	–3.86	<.001	***
Session order	–0.046	0.021	–2.21	.027	*
AoA	–0.776	0.078	–9.94	<.001	***
CONC	–0.037	0.061	–0.608	.543	
FAM	0.525	0.061	8.66	<.001	***
IMG	–0.067	0.063	–1.06	.288	
SemTran	0.194	0.031	6.20	<.001	***

Acc, average accuracy of writing-to-dictation; AoA, age-of-acquisition; CONC, concreteness; FAM, Familiarity; IMG, imageability; SemTran, semantic radical transparency

As expected, higher character frequency, fewer number of strokes, and earlier sessions were associated with higher accuracy. For the character ratings, AoA negatively predicted accuracy, while familiarity and semantic radical transparency positively predicted accuracy. Concreteness and imageability did not significantly predict writing accuracy. Once again, the overall pattern of results followed the predictions of the dual route account of writing-to-dictation of Chinese (Lau, 2021; Weekes et al., 2006). Interestingly, we observed that when all three semantic-related variables were entered into the model, only semantic radical transparency significantly predicted writing-to-dictation accuracy. One possible explanation is that compared with imageability and concreteness, semantic radical transparency better represents the semantic processes involved in writing-to-dictation.

To decide the semantic radical transparency of a character, the semantic features associated with the character and those associated with its semantic radical are compared. The more the two sets of semantic features overlap, the higher is the character’s semantic radical transparency. Therefore, in contrast with the ratings of imageability and concreteness, which considered only the character’s semantic features, the rating of semantic radical transparency requires the considerations of semantic features associated with the target characters and a potential semantic category. For example, while the character 情 [cing4] <emotion> may not be considered highly imageable and concrete in meaning, its meaning is strongly related to the meaning associated with the corresponding semantic radical 忄<feeling-related>. In writing a character, high semantic radical transparency will facilitate selection of the semantic radical and increase the chance of a correct response. Hence, semantic radical transparency may be a more relevant measure of the semantic processes involved in writing-to-dictation of Chinese characters. We suggest that future studies should be conducted to further justify this hypothesis.

The HKCCPN is a comprehensive subjective ratings database of 4376 characters in Cantonese. Overall, the imageability, concreteness, AoA, and familiarity ratings reported here were found to be highly reliable. Their convergent and discriminant patterns in relations to other psycholinguist measures were similar to previous Chinese reports, including those in Cantonese (Wong, 2015), simplified script Mandarin (Cai et al., 2021; Liu et al., 2007; Wang et al., 2020) and traditional script Mandarin (Chang et al., 2016; Chang & Lee, 2020). Beyond these analyses within our database, concurrent and predictive validities of our ratings to other ratings of Chinese characters and behavioral performance were also analyzed. In general, our results showed very high similarity when compared with results reported in previous studies predicting lexical decision, naming, and writing-to-dictation (Chang et al., 2016; Chang & Lee, 2020; Wang et al., 2020).

This database provides novel semantic radical transparency ratings on 3216 compound characters, the largest to our knowledge and a significant expansion relative to previous reports. Our findings showed that the semantic radical transparency ratings were just as highly reliable as our other ratings (split-half *r*^*2*^ = .90, ICC = .89), and that it correlates moderately with other semantic-based variables, namely concreteness (*r* = .48) and imageability (*r* = .48) (Bi et al., 2007; Wong, 2015). This supports the notion claimed by some researchers that the semantic radical, as a sublexical component, activates semantic features during lexical access via the lexical-semantic route (Chen & Weekes, 2004; Law & Yeung, 2010; Law et al., 2005; Wong, 2015). This is further supported by its weak correlations with variables associated with the lexical route (i.e., AoA, familiarity, and number of strokes). Finally, in the predictive validity analysis, we observed that among the three semantic-related variables, only semantic radical transparency significantly predicted writing-to-dictation accuracy. We suggested that this may be because semantic radical transparency is a more relevant measure of the semantic processes involved in writing-to-dictation of Chinese. With the availability of these stimuli, we also call for a deeper understanding of semantic access at the sub-character level within compound characters and individual characters in compound words in studies of morphological processing in Chinese.

In the current study, a within-rater method was used, in which the same 20 raters provided ratings for all variables. Using this method, two kinds of "practice effects" may be present. The first one relates to the repeated practice of using the same scales within the tasks. This practice effect has the advantage of baseline consistency, avoiding the problem of baseline differences when many raters are recruited. Consequently, the obtained results are less noisy and more interpretable. Our achievement of this intended advantage was supported by the observation that the raters became more reliable and produced ratings that had higher agreements in later sessions. On the other hand, a second kind of "practice effect", which concerns the repeated exposures of the same set of stimuli across rating tasks, may also be resulted. The fixed instead of counterbalanced order of rating tasks resulted in increased exposures of the target characters for later tasks. The familiarity domain was rated second to last and indeed, it is observed that the overall familiarity ratings of the data set are relatively high. Although this is expected given the selected targets are the most frequently occurring characters among the 6000+ characters in newspapers (Leung & Lau, 2010), it is unclear whether the relatively high overall familiarity ratings observed in the data set is also partly due to the practice effect described. Overall, we noted both advantages and disadvantages of using a within-rater method. Given the high reliability and validity obtained in this study, we suggest that this within-rater method is a viable option when researchers conduct similar studies in the future. Nevertheless, a counterbalanced or partially randomized order of rating tasks is recommended to minimize the second practice effect described above.

For limitations of the study, we acknowledge that the participant sample is small and limited in diversity and so may not be representative. The ratings are derived from young adults with tertiary education and may not reflect language use from all age ranges of Cantonese speakers nor encompass different Cantonese-speaking demographic populations, such as those that may receive less formal schooling. In addition, we suggest that the ‘number of meanings’ variable may have been too rudimentary and requires further investigation. The number of meanings used was a *type* variable that is not sensitive to which semantic features better represent a particular character. Other computational methods such as latent semantic analysis may provide a better index to quantify the semantic features (Wang et al., 2014). Characters with multiple meanings have more distributed semantic features (e.g., 橫 <horizontal> and <harsh and unreasonable>) rather than one specific meaning, and therefore makes the judgment of its imageability, concreteness, and semantic radical transparency more difficult. We hypothesize that the number of meanings may not necessarily influence ratings of AoA and familiarity, because only the most dominant and immediately available morphemes are needed to rate these variables. In contrast, ratings of concreteness, imageability and semantic radical transparency critically depend on the meaning(s) participants activate and choose to rate on. We recommend that future studies investigating semantic radical transparency should consider the number of meanings or provide contexts to ensure that the intended semantic feature is investigated. Other relevant variables relating to the semantic radical, such as semantic combinability and semantic consistency (Chen & Weekes, 2004), may also constrain the semantic radical transparency effects and should be considered. Lastly, this study did not explicitly differentiate between print AoA and spoken AoA (e.g., Cai et al., 2021). The experimental instructions lean towards the use of spoken AoA, but it remains unclear whether the participants may have based their ratings solely on their print or spoken experience. Despite this, studies that have compared the two kinds of AoA have shown that the two are highly correlated (e.g., Liu et al., 2007) and future studies may try to more systematically distinguish these two kinds of AoA.

To conclude, this study provides 4376 subjective ratings of five lexico-semantic measures (imageability, concreteness, AoA, familiarity, and semantic radical transparency) in Cantonese. Skilled Hong Kong readers gave ratings for all characters and variables across multiple days. We believe the novel inclusion of semantic radical transparency benefits researchers seeking a deeper understanding of the semantic relations in phonetic compound characters and their corresponding sublexical units. Descriptive statistics of the five lexico-semantic measures were presented, and our subsequent analyses verified the reliability of the HKCCPN, as well as its validity with other existing Chinese character databases.

## Appendix

Instructions with English translations of age-of-acquisition (AoA) rating:

在這次任務，我們需要您評定自己第一次學到某些中文字詞的年齡。那就是請你估算，自己初次不論以口頭或書面形式學到個別中文字詞並瞭解其意思的年齡。在後面的頁面，您會看到有一系列中文字，每個字後面都跟有一個年齡量表:

In this task, you’re required to rate the age at which you first acquired the target words and their meanings. In the following pages, a series of target words will be presented. Each presented word will be followed by a rating scale:

你的任務就是給每個文字的首次學習年齡進行評定。當您學得您在7歲時首次學習到所呈現的中文字，就選擇它對應的年齡組 “7–8歲”； 如果您覺得自己在1歲時首次學習到所呈現的中文字，就選擇它對應的年齡組 “0–2歲”。請隨意使用尺規上的所有年齡組別，不必考慮是否使用某個組別多次。

The task requirement is that you’ll have to rate the age-of-acquisition of each presented word. For example, if you first acquire the presented character at the age of 7 years old, select the corresponding age range of 7–8 years. If you first acquire the presented character at the age of 1 year old, select the corresponding age range of 0–2 years. Please feel free to use any of the age range items provided. There is no need to consider whether certain age range items have been repeatedly selected.

例如:

請您按順序填寫每個題目，在填寫過程中，注意不要翻回看前面的選擇。

Please rate each item according to the order of presentation. Do not go back to previous items.

Instructions with English translations of Imageability rating:

有些文字能讓人快速及輕易聯想到圖像或事物，有些則較難。

在後面的頁面，您會看到一系列中文字，每個字後面都跟有一個從1到7的星星量表。它的意義如下:

There are words that can be easily represented by pictures or objects, and there are words that are not as easy. In the following pages, a series of target words will be presented. Each presented word will be followed by a rating scale:

您的任務就是給每個字的「圖像聯想度」進行評定。當您覺得所呈現的中文字的圖像聯想度很高，就選擇它的對應星星數目“7”； 如果這中文字幾乎不能讓你聯想到圖像或事物，那麼說明它的圖像聯想度很低，就選擇它對應的星星數目“1”。“1” 和“7”之間的星星數目表示不同等級的圖像聯想程度。請隨意使用尺規上的所有星星數位，不必考慮是否使用某個星星數目很多次。

The task requirement is that you’ll have to rate the *imageability* of each presented word. For example, if you consider the presented character has very high imageability, select “7” in the scale. If you consider the presented character has very low imageability, select “1” in the scale. The items from “1” and “7” represent the ascending level of imageability. Please feel free to use any of the items provided. There is no need to consider whether certain items have been repeatedly selected.

例如:

請您按順序填寫每個題目，在填寫過程中，注意不要翻回看前面的選擇。

Please rate each item according to the order of presentation. Do not go back to previous items.

Instructions with English translations of Familiarity rating:

在後面的頁面，您會看到一系列中文字，每個字後面都跟有一個從1到7的星星量表。它的意義如下:

In the following pages, a series of target words will be presented. Each presented word will be followed by a rating scale:

您的任務就是給每個字的「熟悉度」進行評定。當您覺得所呈現的中文字的熟悉度很高，就選擇它的對應星星數目“7”； 如果這中文字幾乎不熟悉，那麼說明它的熟悉度很低，就選擇它對應的星星數目“1”。“1” 和“7”之間的星星數目表示不同等級的熟悉度。請隨意使用尺規上的所有星星數位，不必考慮是否使用某個星星數目很多次。

The task requirement is that you’ll have to rate the *familiarity* of each presented word. For example, if you consider the presented character looks highly familiar to you, select “7” in the scale. If you consider the presented character is hardly familiar to you, select “1” in the scale. The items from “1” and “7” represent the ascending level of familiarity. Please feel free to use any of the items provided. There is no need to consider whether certain items have been repeatedly selected.

例如:

請您按順序填寫每個題目，在填寫過程中，注意不要翻回看前面的選擇。

Please rate each item according to the order of presentation. Do not go back to previous items.

Instructions with English translations of Concreteness rating:

那些代表物件、動物、動作或物質等能讓人感受或體驗到的文字都有很高的具體度。 在後面的頁面，您會看到一系列中文字，每個字後面都跟有一個從1到7的星星量表。它的意義如下:

Words that represent pictures, objects, actions, or matter that can be felt or experienced are considered having high *concreteness*. In the following pages, a series of target words will be presented. Each presented word will be followed by a rating scale:

您的任務就是給每個字的「具體度」進行評定。當您覺得所呈現的中文字的具體度很高，就選擇它的對應星星數目“7”； 如果這個字的意思十分抽象，那麼說明它的具體度很低，就選擇它對應的星星數目“1”。“1” 和“7”之間的星星數目表示不同等級的具體度。請隨意使用尺規上的所有星星數位，不必考慮是否使用某個星星數目很多次。

The task requirement is that you’ll have to rate the *concreteness* of each presented word. For example, if you consider the meaning of the presented character is highly concrete, select “7” in the scale. If you consider the meaning of the presented character is highly abstract, select “1” in the scale. The items from “1” and “7” represent the ascending level of concreteness. Please feel free to use any of the items provided. There is no need to consider whether certain items have been repeatedly selected.

例如:

請您按順序填寫每個題目，在填寫過程中，注意不要翻回看前面的選擇。

Please rate each item according to the order of presentation. Do not go back to previous items.

Instructions with English translations of Semantic Radical Transparency rating:

形聲字由形旁和聲旁組成。語義透明度是指一個字與其形旁在含義上的聯繫的密切程度。有些形聲字的意思與其形旁的意思相近(語義透明度高)，有些則兩者意思不相似(語義透明度低)。 在後面的頁面，您會看到一系列中文字與它的形旁，每個字後面都跟有一個從1到7的星星量表。它的意義如下:

Phonetic compounds consist of semantic and phonetic radicals. *Semantic radical transparency* concerns whether the meaning of a target character is related to the meaning of the corresponding semantic radical. Characters sharing similar meanings with their corresponding semantic radicals are considered having high semantic radical transparency. Characters that do not share similar meanings with their corresponding semantic radicals are considered having low semantic radical transparency. In the following pages, a series of target words will be presented. Each presented word will be followed by a rating scale:

您的任務就是給每個字與其形旁的「語義透明度」進行評定。當您覺得所呈現的中文字與其形旁的語義透明度很高，就選擇它的對應星星數目“7”； 如果這中文字的形旁和該中文字的語義幾乎沒有關聯，那麼說明它的語義透明度很低，就選擇它對應的星星數目“1”。“1” 和“7”之間的星星數目表示不同等級的語義透明度。請隨意使用尺規上的所有星星數位，不必考慮是否使用某個星星數目很多次。

The task requires you to rate the *semantic radical transparency* of each presented word. For example, if you consider the semantic radical transparency of the presented character is very high, select “7” in the scale. If you consider the semantic radical transparency of the presented character is very low, select “1” in the scale. The items from “1” and “7” represent the ascending level of semantic radical transparency. Please feel free to use any of the items provided. There is no need to consider whether certain items have been repeatedly selected. 例如:

請您按順序填寫每個題目，在填寫過程中，注意不要翻回看前面的選擇。

Please rate each item according to the order of presentation. Do not go back to previous items.

## Footnotes

1 Typical undergraduate students in Hong Kong are usually fluent in Cantonese, Mandarin and English. But the dominant language used in Hong Kong remains to be Cantonese. According to the results of the Hong Kong 2021 population census, 88.2% of the population aged 5 and over used Cantonese at home (Census and Statistics Department, 2022).

2 In the semantic radical transparency rating experiment, PCs with semantic radicals that have undergone orthographic changes were excluded. For example, according to Xu (1963), when first created, the semantic radical of the PC 虛 [hoey1] <imaginary> was 丘 <jau1> [mound], which has undergone changes orthographically into the bottom part of the character. Since most Chinese readers may not know the original orthographic forms of these targets, asking the participants to judge the semantic radical transparency of the targets in the absence of the semantic radicals in their orthographic forms could deviate from the definition of semantic radical transparency rating. Therefore, these PCs were excluded from the semantic radical transparency rating experiment.
